# Mobile phone ownership, digital technology use and acceptability of digital interventions among individuals on opioid use disorder treatment in Kenya

**DOI:** 10.3389/fdgth.2022.975168

**Published:** 2022-08-25

**Authors:** Sarah Kanana Kiburi, Saeeda Paruk, Bonginkosi Chiliza

**Affiliations:** ^1^ Discipline of psychiatry, Nelson Mandela School of Clinical Medicine, University of KwaZulu-Natal, Durban, South Africa; ^2^ Mbagathi Hospital, Nairobi Metropolitan Services, Nairobi, Kenya

**Keywords:** acceptability, digital interventions, Kenya, mobile phone use, opioid use disorder, treatment

## Abstract

**Background:**

There is limited research on the use of digital interventions among individuals with opioid use disorders (OUD) in low-and-middle income countries. This study aimed to assess mobile phone ownership, digital technology use and acceptability of digital interventions for treatment among individuals on treatment for OUD in Nairobi, Kenya.

**Methods:**

A cross-sectional study was conducted among individuals with OUD. Structured questionnaires were used to collect data on socio-demographic and clinical characteristics, use of mobile phones and other digital technology and acceptability of digital interventions for treatment.

**Results:**

One hundred and eighty participants were enrolled comprising 83.3% males with mean age of 31.5 years (SD 8.6). Mobile phone ownership was reported by 77.2% of participants of which 59.7% used smartphones. One hundred and sixty-six (92.2%) used phones to call, 82.8 and 77.2% used phones to send and receive text messages respectively; 30% used the internet; 57.2% had replaced the phone in past year and 51.1% of participants reported use of at least one social media platform, of these 44.4% had searched social media for information on drug use. Acceptability to receive treatment by phone was 95% and computer 49.4% with majority (88.1%) preferring a text message-based intervention. The preferred approach of delivery of a text message-based intervention were: one text message per day once a week, message to be personalized and individuals allowed to choose time and day to receive the message. Factors associated with acceptability of digital interventions were education level, being single, smartphone ownership and employment.

**Conclusion:**

Majority of individuals on treatment for OUD had access to mobile phones but with high device turnover and limited access to computers and internet. There was high acceptability of digital interventions to provide treatment for OUDs, mostly through phones. These findings highlight factors to consider in the design of a digital intervention for this population.

## Introduction

Opioid use is a public health concern globally as it accounts for the major burden of disease among the substance use disorders (SUDs). This is mainly due to mortality from opioid overdose and other related comorbidities (1, 2). In Kenya opioid use is a growing concern with prevalence rates varying depending on the study population (3). A recent systematic review reported on 21 studies describing opioid use epidemiology in Kenya and the prevalence ranged from 1.1% among individuals on Human Immunodeficiency Virus (HIV) infection treatment to 8.2% among individuals admitted at a psychiatry referral hospital (4). The recommended treatment for opioid use disorder (OUD) is pharmacotherapy in combination with psychosocial treatment (5). Psychosocial treatments that have been traditionally conducted in-person are effective in improving outcomes among people with OUD (6–8). Despite the available treatment for OUD most individuals are not able to access treatment due to a large treatment gap for SUDs globally due to factors such as stigma, poor clinician knowledge and training on OUD, resource restraints and challenges related to national policies on treatment provision (1, 9, 10). This gap is higher in Africa whereby it is estimated that only one in eighteen people with SUD receive treatment (3, 11–13).

Digital interventions involve the use of digital technology and other information technology platforms in delivery of health care services. This can be through mediums such as computers or mobile phones (14, 15). Recently there is growing use of digital technology to deliver psychosocial treatment among individuals with SUD. Digital interventions can address some challenges that contribute to the high treatment gap to improve access to psychosocial treatment for patients with OUD (16). These interventions can offer a more private, convenient, accessible, economical option (17) and help address the stigma associated with in-person attendance for SUD treatment (18, 19). Mobile phones have the added advantage of being more portable compared to other forms of digital intervention platforms and smartphones can be used to connect to internet and run applications for addiction treatment (18, 20).

The increasing use of mobile phones in health care for management of several conditions is attributed to the increase in use of mobile phones (18). In Africa, mobile phone penetration has been increasing with Kenya having a 91% penetration of mobile subscriptions compared to Africa's average of 80% (21). Basic mobile phones are the most common, although the proportion of those with smartphones is increasing. This is based on findings from a 2018 Pew Research that reported only a third of people owned smartphones (22) and a recent nationwide assessment that showed smartphone and feature phones penetration in 2021 as 53.4% and 67.9% respectively (23). In addition, 58% of people reporting no phone ownership share a phone with someone else (22).

In addition, the COVID-19 pandemic required health care providers to review modes of delivery of care for individuals with OUD, with increased use of telemedicine approaches (24–27). A possible challenge is the availability of smartphones and internet access in low and middle income settings, but this can be mitigated by use of text mHealth interventions since basic mobile phones are cheaper and more accessible and have been shown to be effective in delivery of care for patients with SUD (24).

Several studies assessing the use of mobile technology in patients with SUDs have shown high mobile phone ownership and use. For example, a study in United Kingdom among patients in four community treatment facilities reported prevalence of 83% (28); four studies in the United States of America—one among individuals with injection drug use, another among patients at a detoxification Centre, one at an intensive outpatient treatment facility and another at eight SUD treatment centres report prevalence of 66.2%, 86%, 93.8% and 91% respectively (18, 29–31). Individuals with SUD also report access to other digital technology such as computers, internet and social media and willingness to receive SUD treatment using these platforms (30, 32–34). Majority of the participants in these studies accessing digital interventions report that the interventions are helpful and easy to use (14, 35).

Digital interventions have been used for other disorders in Kenya. A systematic review reported on 29 studies assessing mobile phone use in health care and found most studies were focused on HIV, malaria, maternal and child health and one study among patients with hypertension (36). Access to mobile phone ownership varies among the different settings in Kenya. For example a study among youth aged 14–24 years found 64% had access to phones (37); one study among young adults in informal settlements reported prevalence of mobile phones at 79% individual phone ownership and 93% household access whereby 69% were willing to receive HIV counselling *via* phone (38); and a nationwide study in public hospitals reported overall 61.2% phone ownership (39). Two studies report on use and acceptability of telepsychiatry at a private facility. The first was a case study whereby the common telepsychiatry platforms were zoom calls, WhatsApp calls and normal calls while skype was rarely used. Most patients reported to like the intervention (40). The other, a qualitative study among patients and health care providers, reported overall acceptability of the intervention and high perceived effectiveness. Challenges cited by participants included occasional problems with connectivity, privacy concerns and cost (41).

There is limited research on the use of digital interventions among patients with SUDs in low-and-middle income countries (LMICs). In Kenya, only one study has reported on use of digital technology for SUD treatment which comprised single session motivational interviewing *via* mobile phone (42) and one proposed study for peer mHealth intervention among university students (43). According to authors, knowledge, there is no study that describes mobile phone use and acceptability of digital interventions among individuals with OUD in Kenya. To explore the effectiveness of digital interventions among individuals with OUD, it is first important to know the prevalence of mobile phone ownership and other digital technology use and the acceptability of these interventions. Therefore, this study aimed to assess mobile phone ownership, digital technology use and acceptability of digital interventions for SUD treatment and the associated sociodemographic factors among individuals on treatment for OUD.

## Materials and methods

### Study design

This was a cross-sectional descriptive study among individuals with OUD.

### Study setting

This study was carried out at Ngara methadone clinic in Nairobi which is a public clinic that offers methadone treatment to individuals with OUD. The individuals also receive psychosocial support such as psychotherapy and treatment for co-occurring psychiatric disorders. The eligibility criteria for methadone treatment initiation are individuals presenting with OUD as per DSM 5 criteria (44) and testing positive for opioids through urine toxicology screening and are motivated to stop using substances which is confirmed through signing of a treatment consent form.

### Study population

This study included individuals with OUD on methadone treatment. The individuals had to be aged 18 years and older, able to comprehend questions asked in English or Kiswahili languages and willing to provide informed consent. Those who were unable to comprehend the questions and those not able or unwilling to provide consent were excluded.

### Measures

#### Sociodemographic and clinical characteristics of participants

Data on sociodemographic and clinical characteristics of the study participants was collected using a researcher-designed questionnaire which was based on a review of the literature. The sociodemographic variables collected included: age, gender, educational level, marital status and employment. Clinical variables included screening questions for lifetime substance use (substances ever used); current substance use; problem gambling (questions based on brief problem gambling screen (45, 46); questions on exposure to childhood adverse events (physical, sexual or emotional abuse and loss of a parent) and data on current methadone dose and co-occurring medical and psychiatric illness that was extracted from the patients' clinical records.

#### Questions on mobile phone ownership, technology use and acceptability of digital platforms for treatment of substance use disorders

This data was collated using a survey questionnaire which included questions assessing technology use similar to those used in the study by McClure and colleagues (31) modified to include questions on social media use as well as acceptability and interest in using a digital intervention for SUD treatment. Questions on technology use included: mobile phone ownership, smartphone ownership, use of computer, tablets, email, or social media; change of phone in past year and the reasons for the change; and the use of social media to access information on substance use disorders. Technology acceptability refers to users' perception of a system before use, likelihood of using the technology and the extent to which they consider an intervention to be agreeable and satisfactory (47, 48). The digital technologies accessed in our study were mobile phones, computers, tablets, social media use, email and internet access. Digital interventions refer to treatment offered through digital technology platforms. Questions on acceptability of digital interventions included: willingness to receive SUD treatment information on phone, computer, social media, mobile app or online support group; willingness to enroll for a text message intervention and preference in text message intervention delivery such as number of messages per day and week and content of the text messages was also included.

### Recruitment and sampling

Participants were invited to participate during their daily visit to the clinic for methadone. Recruitment was conducted using convenience sampling method. Individuals attending the clinic were informed about the study by the researchers and invited to participate. Those fulfilling the eligibility criteria and willing to participate were asked to sign an informed consent and then given the questionnaire. The questionnaire was a self-administered paper and pencil questionnaire available in English and Kiswahili. Those not able to answer the questions by themselves but could comprehend what was asked, were assisted to fill in the questionnaire by the research assistant.

### Sample size calculation

A sample of 216 participants was calculated using Cochran's (1977) sampling formula, *n = *{*z*^2^ *p* (1−*p*)}/*d*^2^ where ***n*** is estimated sample size; ***d*** is the level of precision; ***p*** is proportion of those with the condition of interest and; z is Confidence level. For this study we used a confidence interval of 95%, expected prevalence of 83% based on previous study (28) and a level of significance of 5% (0.05), which gave a sample size of 216. We corrected sample size for finite population, since the number patients that were currently active on methadone treatment at the clinic during the time of study was 600, and added 10% due to possibility of missing data which gave final sample size of 175. A total of 180 individuals were interviewed for this study.

### Statistical analysis

Descriptive statistics were conducted to estimate the use of technology and acceptability of technology as well as the participant's characteristics. Univariate associations of use and acceptability of technology and other variables were estimated using chi-square tests and Fischer's exact tests in variables where there were low cell counts. Variables with *p* > 0.05 were removed. Multivariate analysis using logistic regression was used to estimate independent predictors of use and acceptability of technology by entering all variables that were associated with the outcome at bivariate level at *p* < 0.1. Adjusted odds ratio with its 95% confidence interval was calculated to report the strength and significance of the association. All tests were two sided and the level of statistical significance was set at *p* < 0.05. There were three outcomes in which multivariate analysis was not possible because of low cell counts and non-significant differences among the covariates (use of text messages; acceptability to receive treatment for SUD *via* phone; and *via* text message).

### Ethical considerations

The study was approved by the University of Nairobi and Kenyatta National Hospital ethics committee (P134/03/2021) and the University of KwaZulu Natal (UKZN) biological research ethics committee (BREC/00002951/2021).

## Results

### Sociodemographic and clinical characteristics of the participants

Table 1 shows a summary of the sociodemographic characteristics. A total of 180 participants were enrolled in the study with a mean age of 31.5 years (SD 8.6), majority were male (83.3%), with a primary school level of education (47.8%). The mean methadone dose prescribed was 54.7 mg (SD 25.8), median was 55.0 mg (range 1–160).

**Table 1:** Sociodemographic characteristics of study participants.

Variable	Category	Frequency (*N* = 180)	Percentage (%)
Gender	Male	150	83.3
	Female	30	16.7
Age (years)	Mean ± SD; Median; Range	31.5 ± 8.6; 30.0; [18–62]	
Age	18–24 Years	42	24.0
	25–40 Years	107	61.1
	40+ Years	26	14.9
	*Non-Response*	5	
Education level	Primary (8 years) and below	86	47.8
	Secondary	65	36.1
	Tertiary	29	16.1
Employment Status	Employed	48	26.8
	Self-Employed	63	35.2
	Unemployed	68	38.0
	*Non-Response*	1	
Monthly personal income in Ksh	<=20,000	150	83.3
	>20,000	30	16.7
Marital status	Married	49	27.5
	Separated/Divorced/Widowed	61	34.3
	Single	68	38.2
	*Non-Response*	2	
Substance use patterns			
Age at first use of any substance	Less than 10 years	15	8.3
	11–15 years	50	27.8
	16–20 years	87	48.3
	21–30 years	23	12.8
	Above 30 years	5	2.8
Lifetime substance use	Opioids	180	100
	Cannabis	164	91.1
	Alcohol	87	48.3
	Smoking	137	76.1
	Khat	67	37.2
	Benzodiazepines	58	32.2
	Others	16	8.9
Current substance use (at time of study)	None	32	17.8
	Opioids	33	18.3
	Cannabis	94	52.2
	Alcohol	12	6.7
	Smoking	66	36.7
	Khat	13	7.2
	Benzodiazepines	7	3.9
	Others	4	2.2
Gambling behaviour in past 12 months	Yes	66	36.7%
Used illicit drug by Injection	Yes	73	40.8
Previous SUD Treatment	Yes	47	26.1
Ever been arrested	Yes	127	70.6
Family History of mental illness	Yes	9	5.0
History of childhood adverse event	Yes	127	70.6
Parent or family member uses substances	Yes	68	37.8
Psychiatry or medical comorbidity	Yes	21	11.7
Duration on methadone treatment	4 Years	33	18.3
	3 Years	51	28.3
	2 Years	30	16.7
	1 Year	25	13.9
	Less than 1 Year	41	22.8

### Mobile phone ownership and technology use among study participants

Table 2 provides a summary of mobile phone ownership and use of technology among the study participants. One hundred and thirty-nine participants (77.2%) reported mobile phone ownership of which 83 (59.7%) were smartphones, comprising 46.1% of total sample. Out of the 41 participants who reported no phone ownership, 28 reported having access to a phone through others (friend *n* = 16, parent *n* = 6, spouse *n* = 4, sibling *n* = 2). One hundred and sixty-six (92.2%) participants used the phone to call and while 58 (32.2%) reported to access social media using phone, 92 (51.1%) reported social media use on any device. One hundred and three participants reported to have changed phones in past year, mainly due to phone getting lost or stolen and of the eleven participants who reported other reasons, nine participants reported selling phone to buy substances, one sold phone to buy food and one person sold to upgrade to a better phone.

**Table 2:** Table showing summary of mobile phone ownership and technology use among participants.

Variable	Category	Frequency (*N* = 180)	Percentage (%)
Own any mobile phone	Yes	139	77.2
	No	41	22.8
Smart Phone (*n* = 139)	Yes	83	59.7
	No	56	40.3
Access to any digital technology platform	Phone	151	83.9
	Computer	45	25.0
	Tablet	12	6.7
	Internet	54	30.0
	None	12	4.4
Purpose/Use of Phone	To Call	166	92.2
	Send SMS	149	82.8
	Receive SMS	139	77.2
	Email	21	11.7
	Browse Internet	63	35.0
	Social media access	58	32.2
Average SMS sent in a week	Not Using	33	18.3
	Less than daily	69	38.3
	Daily	78	43.3
Average SMS received in a week	Not Using	33	18.3
	Less than daily	72	40.0
	Daily	75	41.7
Change of phone in the last one year	Never	77	42.8
	Once	63	35.0
	2–3 times	22	12.2
	More than 3	18	10.0
Reason for change (*n* = 103)	Stolen	43	41.7
	Lost	54	52.4
	Damaged	18	17.5
	Others	11	10.7
Ever received call or text message from clinic staff?	Yes	17	9.4
	No	163	90.6
Use of any social media	Yes	92	51.1
	No	88	48.9
Type of social media (*n* = 92)	WhatsApp	81	45.0
	Facebook	81	45.0
	Instagram	21	11.7
	Twitter	16	8.9
	YouTube	12	6.7

### Use of social media to search information regarding substance use disorders

Eighty participants (44%) reported using social media to search for information about substance use and SUD and 59 (32.8%) had seen drug cues (things that made one want to use substances) while using social media. Data on social media use on substance use and recovery is summarised in Table 3.

**Table 3:** Summary of social media use to seek substance use disorder-related information.

Variable	Category	Frequency (*N* = 180)	Percentage (%)
Ever used your phone to search for information about substance use problems	Yes	80	44.4
	No	100	55.6
Type of information (*n* = 80)	Types of substances	24	30.0
	Harms associated with substance use	54	67.5
	Treatment of substance use disorder	59	73.8
	Recovery support groups	21	26.3
	Others	2	2.5
Seen recovery information on social media	Always	6	3.3
	Many times	26	14.4
	A few times	55	30.6
	Never	93	51.7
Ever posted information in social media about being in recovery	Yes	23	12.8
	No	157	87.2
Ever seen drug cues on social media	Always	5	2.8
	Many times	11	6.1
	A few times	43	23.9
	Never	121	67.2

### Acceptability of digital interventions for substance use disorder treatment

One hundred and seventy-one (95%) participants were willing to receive SUD treatment through mobile phone and 89 (49.4%) through use of computers. Table 4 shows a summary of the acceptability and preference for use of digital technology for SUD treatment among the study participants.

**Table 4:** Acceptability of digital technology for substance use treatment.

Variable	Category	Frequency (*N* = 180)	Percentage (%)
Willing to receive substance use disorder treatment through phone	Yes	171	95.0
Willing to receive substance use disorder treatment through computer	Yes	89	49.4
Consider social media a good place to receive information to help you stop using substances	Yes	91	50.6
Would join an online support group during treatment	Yes	82	45.6
Treatment preference	Text Message	144	84.2
	Voice Call	141	82.5
	WhatsApp Group	52	30.4
	Smartphone app	37	21.6
	Internet/Website	22	12.9
	Facebook Page	20	11.7
	Email	6	3.5
Preference for treatment delivery	Individual	100	58.8
	In a group	17	10.0
	Both	53	31.2
	Non-Response	10	
Willing to use an app on phone to help on recovery from substance use	Yes	86	48.9
Willing to sign up to receive text messages to help during treatment/recovery?	Yes	156	88.1
Frequency of text messages in a week (*n* = 156)	Once a week	62	39.7
	2–3 times in a week	45	28.8
	4–5 times in a week	14	9.0
	Daily	35	22.4
Frequency of text messages per day (*n* = 156)	One	96	61.5
	2	27	17.3
	3–5	21	13.5
	>5	12	7.7
Preference for the text message content (*n* = 156)	Message to be personalized different for each individual	62	39.7
	Same message to be sent to all those receiving the treatment	50	32.1
	Both approaches combined	44	28.2
Preference for the time to receive text message (*n* = 156)	Text message sent randomly at any day of the week	40	25.6
	Text message sent randomly at any time of the day	27	17.3
	Text message sent in the evening	36	23.1
	Choose the time and day to receive the text message	53	34.0

### Factors associated with mobile phone ownership and digital technology use

Tables 5, 6 illustrates the factors associated with mobile phones and digital technology use on bivariate and multivariate analysis respectively.

**Table 5:** Factors associated with mobile phone ownership and other digital use on bivariate analysis.

Variable	Category	Phone ownership *n* (%)	Chi square *p* value	Smart phone *n* (%)	Chi square *p* value	Access to any digital media *n* (%)	Chi square *p* value	Use of text message		Chi square *p* value	Use of social media *n* (%)	Chi square *p* value
								Less than daily *n* (%)	Daily *n* (%)			
Gender	Male	115 (76.7)	0.691	66 (44.0)	0.204	140 (93.3)	1.000	59 (39.3)	62 (41.3)	0.459	74 (49.3)	0.286
	Female	24 (80.0)		17 (56.7)		28 (93.3)		10 (33.3)	16 (53.3)		18 (60.0)	
Age (years)	18–24	31 (73.8)	0.559	19 (45.2)	0.997	41 (97.6)	0.074	20 (47.6)	16 (38.1)	0.418	22 (52.4)	0.902
	25–40	81 (75.7)	–	49 (45.8)	–	96 (89.7)	–	36 (33.6)	47 (43.9)	–	53 (49.5)	
	40+	22 (84.6)	–	12 (46.2)	–	26 (100.0)	–	11 (42.3)	12 (46.2)	–	14 (53.8)	
Education level	Primary and below	54 (62.8)	**<0.001**	23 (26.7)	**<0.001**	78 (90.7)	0.217	32 (37.2)	27 (31.4)	**<0.001**	28 (32.6)	**<0.001**
	Secondary	56 (86.2)	–	35 (53.8)	–	61 (93.8)	–	28 (43.1)	31 (47.7)	–	37 (56.9)	
	Tertiary	29 (100.0)	–	25 (86.2)	–	29 (100.0)	–	9 (31.0)	20 (69.0)	–	27 (93.1)	
Employment Status	Employed	44 (91.7)	–	35 (72.9)	**<0.001**	46 (95.8)	0.190	15 (31.3)	29 (60.4)	**0.039**	35 (72.9)	**0.001**
	Self-Employed	48 (76.2)	**0.015**	26 (41.3)		61 (96.8)	–	23 (36.5)	27 (42.9)	–	31 (49.2)	
	Unemployed	47 (69.1)		22 (32.4)		61 (89.7)	–	31 (45.6)	22 (32.4)	–	26 (38.2)	
Income	<=20,000	112 (74.7)		60 (40.0)	**<0.001**	139 (92.7)	0.423	61 (40.7)	59 (39.3)	0.051	67 (44.7)	**<0.001**
	>20,000	27 (90.0)	0.068	23 (76.7)	–	29 (96.7)	–	8 (26.7)	19 (63.3)		25 (83.3)	
Civic Status	Married	40 (81.6)		29 (59.2)	**0.054**	46 (93.9)	0.854	20 (40.8)	24 (49.0)	0.449	37 (75.5)	**<0.001**
	Separated/Divorced/Widowed	47 (77.0)	0.590	22 (36.1)	–	56 (91.8)	–	25 (41.0)	23 (37.7)	–	21 (34.4)	
	Single	50 (73.5)		31 (45.6)		64 (94.1)		24 (35.3)	29 (42.6)		33 (48.5)	
Current Substance use	None	29 (90.6)	**0.046**	19 (59.4)	0.097	32 (100)	0.095	14 (43.8)	16 (50.0)	0.143	22 (68.8)	**0.028**
	Yes	110 (74.3)		64 (43.2)		136 (91.9)	–	58 (39.2)	59 (39.9)		70 (47.3)	
Phone Ownership	No	–	–	–	–	–	–	–	–	–	3 (7.3)	**<0.001**
	Yes	–	–	–	–	–	–	–	–	–	89 (64.0)	
Smart Phone Ownership	No	–	–	–	–	–	–	–	–	–	11 (11.3)	**<0.001**
	Yes	–	–	–	–	–	–	–	–	–	81 (97.6)	

**Table 6:** Factors associated with mobile phone ownership and other digital technology use on multivariate analysis.

Variable	Reference category	Mobile phone ownership		Smartphone ownership		Use of social media	
		aOR (95%CI)	*p* value	aOR (95%CI)	*p* value	aOR (95%CI)	*p* value
Education	Secondary versus primary	2.92 (1.24–6.89	0.015	2.37 (1.11–5.7)	0.026	0.44 (0.08–2.57)	0.363
	Tertiary versus primary			11.23 (3.32–38.04)	0.000	1.92 (0.14–26.26)	0.624
Employment status	Employed versus unemployed	2.76 (0.80–9.50)	0.108	3.76 (1.47–9.65)	0.006	0.32 (0.04–2.73)	0.296
	Self-employed versus unemployed	1.24 (0.54–2.85)	0.618	1.29 (0.57–2.95)	0.541	1.79 (0.34–9.49)	0.493
Income level	Above Ksh 20,000 versus Below Ksh20,000	1.64 (0.42–6.50)	0.479	3.05 (1.05–8.85)	0.040	8.64 (0.77–96.54)	0.080
Marital status	Separated versus married	–		0.56 (0.23–1.39)	0.212	0.01 (0.00–0.16)	0.001
	Single versus married	–		1.04 (0.42–2.55)	0.936	0.08 (0.01–0.51)	0.007
Mobile phone ownership	Mobile phone versus no phone	–		–		3.07 (0.50–18.94)	0.264
Smartphone ownership	Smartphone versus no smartphone	–		–		1769 (86–36,390)	<0.001

Mobile phone ownership was significantly associated with employment status, education and current substance use on bivariate analysis. On multivariate analysis the significant factor was education whereby those with secondary level education were more likely to report phone ownership. Factors associated with smartphone ownership on bivariate analysis were education, employment status, income level and marital status. On multivariate analysis both secondary and tertiary level of education, being employed and in the higher income category increased odds for smartphone ownership.

Access to any digital technology was not significantly associated with any sociodemographic characteristic. Factors associated with use of text messages on bivariate analysis were employment, education and income level. Multivariate analysis was not possible for this subcategory. Factors associated with use of social media on bivariate analysis were education level, employment status, income, marital status, current substance use, phone ownership and smartphone ownership. On multivariate analysis marital status was significant whereby those separated and single were less likely to use social media and those with smartphones had higher likelihood of social media use.

### Factors associated with acceptability of digital interventions for SUD treatment

Tables 7, 8 provide a summary of factors associated with acceptability of various digital interventions for substance use disorder treatment on bivariate and multivariate analysis respectively.

**Table 7:** Factors associated with acceptability of using digital technology in substance use disorder treatment on bivariate analysis.

Variable	Category	Willing to use phone *N* (%)	Chi square *p* value	Willing to use computer *N* (%)	Chi square *p* value	Would join online platform *N* (%)	Chi square *p* value	Willing to use social media *N* (%)	Chi square *p* value	Willing to use mobile app *N* (%)	Chi square *p* value	Willing to use text message *N* (%)	Chi square *p* value
Gender	Male	144 (96.0)	0.169	70 (46.7)	0.096	69 (46.0)	0.789	73 (48.7)	0.257	72 (48.0)	0.894	131 (87.3)	0.556
	Female	27 (90.0)		19 (63.3)		13 (43.3)		18 (60.0)		14 (46.7)		25 (83.3)	
Age	18–24 Years	40 (95.2)	0.981	16 (38.1)	0.240	17 (40.5)	0.783	19 (45.2)	0.683	15 (35.7)	0.186	35 (83.3)	0.738
	25–40 Years	102 (95.3)		57 (53.3)		50 (46.7)		56 (52.3)		56 (52.3)		94 (87.9)	
	40+ Years	25 (96.2)		12 (46.2)		12 (46.2)		12 (46.2)		12 (46.2)		23 (88.5)	
Education level	Primary and below	78 (90.7)	**0** **.** **038**	26 (30.2)	**<0.001**	25 (29.1)	**<0** **.** **001**	31 (36.0)	**<0** **.** **001**	25 (29.1)	**<0** **.** **001**	66 (76.7)	**0** **.** **001**
	Secondary	64 (98.5)		39 (60.0)		33 (50.8)		35 (53.8)		35 (53.8)		61 (93.8)	
	Tertiary	29 (100.0)		24 (82.8)		24 (82.8)		25 (86.2)		26 (89.7)		29 (100.0)	
Employment Status	Employed	48 (100.0)	0.064	31 (64.6)	**0.031**	26 (54.2)	0.397	29 (60.4)	0.299	30 (62.5)	**0** **.** **036**	45 (93.8)	0.265
	Self-Employed	61 (96.8)		31 (49.2)		27 (42.9)		30 (47.6)		30 (47.6)		54 (85.7)	
	Unemployed	62 (91.2)		27 (39.7)		29 (42.6)		32 (47.1)		26 (38.2)		57 (83.8)	
Income	Above Ksh. 20,000	144 (96.0)	0.169	66 (44.0)	**0.001**	65 (43.3)	0.181	73 (48.7)	0.257	66 (44.0)	**0** **.** **023**	130 (86.7)	1.000
	Below Ksh. 20,000	27 (90.0)		23 (76.7)		17 (56.7)		18 (60.0)		20 (66.7)		26 (86.7)	
Civic Status	Married	44 (89.8)	0.120	34 (69.4)	**0.003**	32 (65.3)	**0** **.** **002**	33 (67.3)	**0** **.** **008**	32 (65.3)	**0** **.** **012**	44 (89.8)	0.638
	Separated/Divorced/Widowed	60 (98.4)		28 (45.9)		27 (44.3)		31 (50.8)		27 (44.3)		51 (83.6)	
	Single	65 (95.6)		26 (38.2)		22 (32.4)		26 (38.2)		26 (38.2)		59 (86.8)	
Current Substance use	None	31 (96.9)	0.591	17 (53.1)	0.646	18 (56.3)	0.180	20 (62.5)	0.136	19 (59.4)	0.148	29 (90.6)	0.468
	Yes	140 (94.6)		72 (48.6)		64 (43.2)		71 (48.0)		67 (45.3)		127 (85.8)	
Phone Ownership	No	37 (90.2)	0.112	6 (14.6)	<0.001	6 (14.6)	**<0** **.** **001**	7 (17.1)	**<0** **.** **001**	5 (12.2)	**<0** **.** **001**	26 (63.4)	**<0** **.** **001**
	Yes	134 (96.4)		83 (59.7)		76 (54.7)		84 (60.4)		81 (58.3)		130 (93.5)	
Smart Phone Ownership	No	91 (93.8)	0.430	25 (25.8)	<0.001	21 (21.6)	**<0** **.** **001**	27 (27.8)	**<0** **.** **001**	22 (22.7)	**<0** **.** **001**	77 (79.4)	**0** **.** **002**
	Yes	80 (96.4)		64 (77.1)		61 (73.5)		64 (77.1)		64 (77.1)		79 (95.2)	

**Table 8:** Factors associated with acceptability of using digital technology in substance use disorder treatment on multivariate analysis.

Variable	Reference category	Social media good place for SUD treatment		Would join online support group for SUD treatment		Willing to receive SUD treatment *via* computer		Use an app to receive treatment on recovery	
		aOR (95% CI)	*p* value	aOR (95% CI)	*p* value	aOR (95% CI)	*p* value	aOR (95% CI)	*p* value
Education	Secondary versus primary	1.01 (0.47–2.21)	0.974	1.32 (0.59–2.91)	0.499	2.02 (0.90–4.50)	0.086	0.13 (0.03–0.53)	0.005
	Tertiary versus primary	4.00 (1.09–14.66)	**0.037**	4.47 (1.28–15.61)	**0.019**	3.36 (0.94–11.28)	0.063	0.20 (0.05–0.84)	**0.028**
Employment status	Employed versus unemployed	–		–		0.83 (0.30–2.29)	0.724	0.93 (0.33–2.61)	0.893
	Self-employed versus unemployed	–		–		1.26 (0.53–3.01)	0.598	1.26 (0.52–3.07)	0.606
Income level	Above Ksh 20,000 versus below Ksh 20,000	–		–		2.12 (0.71–6.34)	0.179	1.13 (0.38–3.31)	0.828
Marital status	Separated versus married	0.84 (0.33–2.14)	0.719	0.71 (0.28–1.81)	0.470	0.52 (0.20–1.38)	0.189	0.71 (0.27–1.88)	0.489
	Single versus married	0.29 (0.12–0.75)	0.011	0.23 (0.09–0.61)	**0.003**	0.27 (0.10–0.73)	**0.009**	0.32 (0.12–0.85)	**0.023**
Mobile phone ownership	Mobile phone versus no phone	2.41 (0.87–6.69)	0.092	1.80 (0.60–5.39)	0.296	2.69 (0.89–8.17)	0.081	0.42 (0.13–1.32)	0.136
Smartphone ownership	Smartphone versus no smartphone	5.31 (2.32–12.15)	**<0.001**	6.65 (2.84–15.57)	**<0.001**	4.93 (2.07–1175)	**<0.001**	0.16 (0.06–0.38)	**<0.001**

Factors associated with acceptability of SUD treatment through computer on bivariate analysis were education level, employment status, income level, marital status, mobile phone ownership and smartphone ownership. On multivariate analysis, being single was associated with low acceptability and those with smartphone ownership were more likely to accept treatment through computers.

Factors associated with acceptability to receive SUD treatment through social media on bivariate analysis were education level, marital status, phone ownership and smartphone ownership. On multivariate analysis, those with tertiary level of education and had a smartphone were more likely to use social media for treatment while being single reduced the likelihood to accept treatment through social media.

Factors associated with acceptability of joining online support group for SUD treatment on bivariate analysis were education, marital status, mobile phone ownership and smartphone ownership. On multivariate analysis factors tertiary level education and smartphone ownership were associated with increased odds of joining an online group while being single was associated with reduced acceptability.

Factors associated with acceptability to receive substance use treatment through phone on bivariate analysis was education level. Multivariate analysis was not done for this subcategory. Factors associated with acceptability of text message for SUD treatment on bivariate analysis were education level, phone ownership and smartphone ownership. Multivariate analysis was not possible for this subcategory.

Factors associated with acceptability to use an app to receive SUD treatment on bivariate analysis were education level, employment status, income level, marital status, phone ownership and smart phone ownership. On multivariate analysis factors with associated with use of an app for SUD treatment were: education level, whereby secondary and tertiary level of education were associated with reduced likelihood of using an app (Table 8).

## Discussion

This study assessed mobile phone ownership, use of digital technology and acceptability of digital interventions for SUD treatment among individuals with OUD. Overall, there was high mobile phone ownership, high phone turnover rate, lower levels of social media engagement, variable acceptability for digital interventions in treatment with majority preferring phone calls and text message. In addition, participants gave their preference for a design of a text message-based intervention. Acceptability of digital interventions was associated with education level, being single, smartphone ownership and employment.

Mobile phone ownership was high with 77.2% of participants reporting mobile phone ownership of which 46.1% were smartphones. This is similar to findings of the study among youth whereby 48% had smartphones (37) and high mobile phone penetration in the general population (21, 23). This is also consistent with other studies where phone ownership among individuals with OUD ranges between 77%–89% (28, 33, 34). Access to other digital technologies was lower with 25% and 30% reporting access to computer and internet respectively. This suggests that for a digital intervention for this population, use of a mobile phone-based approach will be most feasible and have more reach compared to those that require internet or computer access.

In this study majority of participants reported using phones to call and send text message. Only 18.3% reported no use of text messages and majority (43.3%) used text message daily. Half of participants (51.1%) reported use of social media. This is slightly lower but in keeping with other studies among individuals with SUD whereby text messages use is reported to range from 91%–94.3% (28, 32, 34). The low social media engagement found in this study is in contrast to findings in high income countries where social media use is reported to range from 67.9% to 79.2% (18, 32). This may be due to low access to internet as reported by 30% of participants in our study compared to up to 96.2% daily internet use in studies in high income countries (32). This is more consistent with the average social media use in our setting as shown by findings from a study among youth attending general outpatient in Kenya where 55% reported access to social media and 22.3% had weekly access to internet (37).

There was a high phone turnover with 57.2% reporting to have changed phone at least once in past year mainly due to phones getting lost and stolen which is a pattern common in individuals with SUD (28, 31, 33, 34). This implies a potential challenge when assessing the effectiveness of an intervention over a follow up period. However this can be mitigated by clinicians at treatment facilities regularly updating contact details of those on treatment (28). There is also possibility of breach of privacy if another person gets access to the patient's information. Potential measures to mitigate the issue of privacy include; having password protection, encryption of messages where possible and relaying only information that does not infringe on confidentiality (28).

Almost half of participants (44%) reported using social media to search information about SUD and mostly information on treatment and online recovery support groups. While only 12.8% had ever posted information about being in recovery on social media, 49.3% reported to have seen information to help them in recovery while using social media. Patients with SUDs search information on general health and addiction-related information (18, 32, 34). Although some studies have found that participants reported difficulty in navigating through social media to search for information, majority of participants report that they understand the content provided online pertaining SUD recovery or general health information (30). This finding shows that social media may be a viable option to provide information on SUD treatment. The limited sharing of recovery information on social media (only 12.8% ever posted about being in recovery) may be related to concerns about stigma which are still very prevalent in different societies based on variables such knowledge, ethnicity and culture (49). Among individuals with SUD, secrecy is reported as a way to cope with perceived stigma to avoid negative consequences such as effect on employment (50).

We observed that a third of participants (32.8%) had seen drug cues on social media which shows a potential harm of digital technology. This has been reported in previous studies whereby in one study 67.5% reported to have seen drug cues on social media (32) while in another study 38% reported to use online platforms to locate drug dealers and get information on how to use substances (30). This implies that increased use of social media could be a potential harm among individuals with SUD hence a need to include this as part of psychoeducation during the implementation of an intervention and teach participants skills to deal with drug cues if they appear. This also provides an opportunity that should be explored to provide digital interventions for SUD treatment to increase the probability of someone accessing information on SUD recovery, immediately after seeing drug cues online to reduce recurrence of use (32).

Phone ownership and use of other digital technology was associated education level, having access to a smartphone, being employed and high-income category. This pattern is similar to previous studies (18, 28) although some studies have reported higher technology use among those younger which was not observed in our study (32). This reflects the sociodemographic factors that need to be considered during design of a digital intervention for treatment in this population. While there are measures that have been used counter the sociodemographic differences on phone use such as providing participants with airtime for research, use of reverse call charging, use text message packages that are cost effective and provision of free internet at the clinic for the participants to access treatment (32), this may not be cost-effective for long-term treatment.

Almost all participants (95%) were willing to receive SUD treatment through mobile phone, 49.4% through use of computers and 45.6% would join an online support group. This is similar to findings in a study in India where 79.3% were willing to use mobile phones to manage craving, 80% were interested in receiving text message for OUD treatment, 64% willing to download apps and use to monitor substance use (34). In another study 86% were willing to be contacted by phone by a health care provider (28). This implies that digital interventions can be used to offer treatment for individuals with opioid use disorders.

Majority of the participants in this study preferred to receive treatment *via* text message and voice calls with less than a tenth preferring email for digital treatment of SUDs. This is supported by other studies in similar populations where participants reported that they would prefer to sign up for text message (18, 34). This may be explained by the low number reporting access to internet and majority reporting to use phones to call and send text messages in this study.

Among those who reported that they would sign up for a text message intervention to support them in treatment, majority preferred to receive a text message once a week and only one text message per day. While 39.7% preferred the message to be personalised for each individual, 32.1% preferred same message sent to those in treatment and a third preferred to choose time and day to receive the text message intervention. A similar pattern was reported by Milward and colleagues (28) with 36% of participants expressing interest in choosing time of day to receive the messages and 35% preferred to choose the frequency of message. This is again important to consider during design of text message interventions to improve engagement and adherence to the intervention. A limitation that may arise with use of text message for SUD treatment is lack of physical and non-verbal cues seen in in-person treatment. Strategies that can improve effectiveness of text message intervention include use of tailored and personalized messages that can include links to initiate a series of messages depending on individual needs of patients (51–53), using a behaviour change theory in the intervention (51, 52), having an interactive program where participants can ask or respond to questions and combining the text message intervention on with in-person sessions (51) and involving the participants in design of the intervention so as to get their preferences in design of the intervention (52, 54).

Factors associated with acceptability and willingness to use digital interventions for SUD treatment were education level with people with tertiary level of education being more likely to use social media or join an online support group and smartphone phone ownership increased odds for reporting acceptability of treatment through computer, social media or online support group as seen in previous studies (30–32). Being single was associated with low acceptability for using computer, social media and online support group. This can be due to the findings that majority of participants in this study were single and being single was associated with low social media use. This can also be related to the finding that marital status and relationships have an influence in substance use behaviour and treatment outcomes (55, 56) that involvement of partners in treatment is associated with better outcome (57, 58). This has practice implications to guide design of digital interventions in order to have an approach that considers the sociodemographic difference among individuals to ensure inclusion. However, more research is needed to explain this further.

In this study, the unexpected results were the factors associated with use of an app for treatment where secondary and tertiary education level and smartphone ownership was associated with reduced odds of acceptability. This is in contrast to other studies and a possible explanation may be limited sample size as due to low number of individuals reporting acceptability of using an app (48.9%) compared to other studies. It may also be possible that the participants may not have understood what an app is when responding to the question or have difficulty accessing apps. This will need to be explored further.

### Strengths and limitations

The strength of this study is that it is first study in Kenya to document mobile phone ownership, use of digital technology and acceptability of digital technology for treatment among individuals with OUD hence providing valuable data in this topic from a low-and middle-income country (LMIC) setting.

Limitations for this study include that firstly this was a cross-sectional study hence it is not possible to determine causal relationships. Second, the information provided was based on self-report which could have had biases such as recall bias or social desirability leading to over or under-reporting of some information. Third, the study was among individuals with opioid use disorder on methadone, at one treatment facility, hence limited generalizability to other populations with other substance use disorders.

## Conclusion

The study findings showed that majority of individuals on treatment for OUD had access to mobile phones and less access to computers and internet. In addition, there was high acceptability of use of digital interventions to provide treatment for substance use disorders mostly through phones *via* text messages. In addition, findings reveal preferences for design of text message intervention for the individuals and sociodemographic such as education and age factors may influence digital technology use patterns.

These findings have several implications for practice. First, it shows that use of digital interventions that involve mobile phones through text messaging may be feasible in the study population. However, the high phone turnover points to a possible challenge that those designing mHealth interventions among individuals with opioid use disorders need to consider and have strategies in place to address this during delivery of the intervention. Second, the high acceptability of the digital approach to provide substance use treatment, shows that this is an approach that can be explored to improve treatment outcomes.

Recommendation for further research include involving qualitative methods to further assess the factors associated with digital intervention use among individuals with opioid use disorders. Further research is also needed to pilot digital interventions among the study population to assess the feasibility of this in an LMIC setting since most studies on digital interventions among individuals with opioid use disorder have been done in high income countries.

## Data Availability

The raw data supporting the conclusions of this article will be made available by the authors, without undue reservation.
